# Seven 365-Million-Year-Old Trilobites Moulting within a Nautiloid Conch

**DOI:** 10.1038/srep34914

**Published:** 2016-10-05

**Authors:** Rui-Wen Zong, Ruo-Ying Fan, Yi-Ming Gong

**Affiliations:** 1 State Key Laboratory of Biogeology and Environmental Geology, School of Earth Sciences, China University of Geosciences, Wuhan, 430074, China

## Abstract

A nautiloid conch containing many disarticulated exoskeletons of *Omegops cornelius* (Phacopidae, Trilobita) was found in the Upper Devonian Hongguleleng Formation of the northwestern margin of the Junggar Basin, NW China. The similar number of cephala, thoraces and pygidia, unbroken thoraces, explicit exuviae, and lack of other macrofossils in the conch, indicate that at least seven individual trilobites had moulted within the nautiloid living chamber, using the vacant chamber of a dead nautiloid as a communal place for ecdysis. This exuvial strategy manifests cryptic behaviour of trilobites, which may have resulted from the adaptive evolution induced by powerful predation pressure, unstable marine environments, and competition pressure of organisms occupying the same ecological niche in the Devonian period. The unusual presence of several trilobites moulting within a nautiloid conch is possibly associated with social behaviours in face of a serious crisis. New materials in this study open a window for understanding the survival strategy of marine benthic organisms, especially predator-prey interactions and the behavioural ecology of trilobites in the middle Palaeozoic.

As one of the major groups in the Palaeozoic marine environments, trilobites have been studied extensively with regard to their systematic palaeontology, biostratigraphy and palaeoecology, based on nearly 300 years of worldwide research. Preliminary understanding exists on the biotic activity (such as breeding, moulting, feeding) and ontogeny of trilobites^1^,^2^. However, there are few advances in the study of the individual and social behaviours and survival strategy of trilobites, perhaps due to the scarcity of fossils that can reflect such processes. The cryptic behaviour of trilobites is an intriguing form of behaviour, which arose in the Cambrian^3^, mainly among tiny trilobites, such as *Pagetia* and *Skreiaspis*, and also agnostids that hid within the tubes of hyoliths and priapulids, or the exoskeletons of large polymerid trilobites^3^,^4^,^5^,^6^,^7^. Since the Ordovician, increasing numbers of predators, as well as the explosion of other competitive organisms that occupied similar ecological niches to trilobites, resulted in increased survival pressure on trilobites. They gradually lost their Cambrian predominance, and recessed since the middle Palaeozoic, when more prominent cryptic behaviour and survival strategy started to develop. In order to avoid the attacks of predators and the stresses of marine environments, they chose the conchs of other invertebrates as shelters^8^,^9^,^10^, or hid in the burrows of other creatures^3^,^11^, and some others may even have excavated open tunnels in the seafloor for self-protection^12^.

In addition to escaping the attacks of predators and/or the harsh environments, another important reason for the cryptic behaviour was to ensure they were safe during critical life activities, such as moulting. Trilobites need a quiet environment to complete ecdysis, such as deeper seawater, quiet waters and without interference from other organisms^13^,^14^, and this process might well have been lethal if trilobites suffered any interference from other creatures or from stresses in the external environment during moulting^15^,^16^. Exuviation of trilobites has been reported frequently, and different modes and techniques employed in this process have been proposed^13^,^15^,^17^,^18^,^19^, but the behavioural strategy of trilobites during moulting is still not well known, due to the rarity of such instances in the fossil record. To date, only a few specimens are available, showing trilobites moulting within the conchs or burrows of other creatures, or moulting within the sea-floor sediments (infaunal moulting)^3^,^9^,^20^,^21^. In the context of powerful predation and competition pressures, as well as unstable marine environment in the middle Palaeozoic, it is perhaps a little surprising that trilobites so often moulted successfully. Here we present an exceptional preservation of several exuviae of *Omegops cornelius* (Phacopida, Phacopidae) emplaced in a nautiloid conch from the Upper Devonian Hongguleleng Formation of the northwestern margin of the Junggar Basin, Xinjiang (Fig. 1), which provides new data for further understanding the moulting behaviour and survival strategy of trilobites in the middle Palaeozoic.

## Results

The weathered nautiloid conch was collected from the bioclastic limestones of storm origin in the Late Devonian Hongguleleng Formation. With only an incomplete living chamber and 4 septa (Fig. 2A), the nautiloid cannot be identified to the generic level. Fragments of *Omegops cornelius* are found in the living chamber, including cephala, pygidia, and some isolated and articulated thoracic segments. In these sclerites, the number of cephala, thoraces and pygidia is almost the same, namely 7 cephala, 7 thoraces and 4 pygidia (Fig. 2A–E). Except for a single thorax that is articulated with a pygidium, all the sclerites are scattered with respect to each other. The way-up of cephala, thoraces and pygidia lies in different directions, i.e. upward, overturned, or vertical and oblique to the longitudinal section of the nautiloid conch (Fig. 2C,E). Some enrolled thoraces consist of all eleven thoracic segments. In addition, all sclerites are crowded or superposed together, and there are more sclerites situated close to the septum and conch-wall (6 cephala, 4 thoraces and 3 pygidia) than those in the central part (1 cephalon, 3 thoraces and 1 pygidium) of the living chamber (Fig. 2A–C,E).

The scattered appearance of *Omegops* without any articulated exoskeletons may be at first attributed to post-mortem transportation by bottom currents in the storm-influenced environment. Outside the nautiloid conch, there are many allochthonous fragments of trilobites preserved in the storm-influenced bioclastic limestones, that are characterized by scattered cephala, pygidia and thoracic segments (Fig. 2L), but there is a lack of articulated thoraces, or there may be only many convex-up cephala gathered in clusters (Fig. 2K). However, the sclerites buried within the nautiloid conch are obviously different from those preserved outside, in terms of the similar number of cephala, thoraces and pygidia, unbroken thoraces, as well as the random orientation of the cephala, thoraces and pygidia. Furthermore, numerous brachiopods, bryozoans, crinoids and other macrofossils are preserved in sediments around the nautiloid conch, some together with sclerites of *Omegops* (Fig. 2L), but there are no other macrofossils except *Omegops* within the nautiloid conch, even though the sizes of some fossils are less than or close to the dimensions of the trilobite sclerites. It is most unlikely that only *Omegops* was transported into the nautiloid conch if they were allochthonous sclerites. In conclusion, herein we regard these sclerites of *Omegops* as autochthonous fossils.

By so stating, we have excluded the possibility that these trilobites are food residues of the hosting nautiloid. Cephalopods are carnivorous animals, and both extant nautilus and Mesozoic ammonites have strong jaws and radulas^22^,^23^. Food residue containing broken crustaceans and bivalves has been reported in some Mesozoic ammonite conchs^23^,^24^. In our case, however, there are no any bite marks or sclerite fractures, thus it excludes the likelihood of being food residue. As a result, these autochthonous sclerites perhaps belong to the corpses or exuviae of *Omegops*, but the latter is more likely, because they are explicitly exuvial sclerites rather than articulated exoskeletons in the conch. In the sclerites shown in Fig. 2F–H, the thorax is articulated with the pygidium, but the cephalon is separated, and situated in one side with distinct rotation. This accords with the moulting pattern of Phacopidae that lack functional facial sutures, where the ecdysial suture is produced between the cephalon and thorax, and the trilobites emerge forward by pushing the cephalon away from the body (inversion or rotation) during the ecdysial process. Such technique has been documented in many phacopid trilobites^13^,^17^,^25^, which was also frequently found in other *Omegops* preserved *in situ* from the argillaceous limestones of the Hongguleleng Formation (Fig. 2I,J).

## Discussion

### Ecdysial process of *Omegops* within the nautiloid conch

Under certain ecological conditions, trilobites will adopt particular strategies to protect themselves from harm during ecdysis. For example, some trilobites can burrow into the seafloor sediments for moulting^21^, or moult within the conchs or burrows of other marine invertebrates^3^,^9^,^11^,^20^. Our specimen is unusual because there are exuviae of at least 7 trilobite individuals buried in the nautiloid conch. Because of the restricted space of the living chamber, as well as the strict environmental requirements of moulting trilobites (no disturbance from other creatures), it is unlikely that these trilobites moulted simultaneously. In other words, the living chamber was either the individual space of a single *Omegops* for repetitive moulting, or a communal space wherein different *Omegops* moulted. Trilobites grow by ecdysis like numerous modern arthropods^2^, and they can use other vacant chambers shed by other animals as places for moulting, e.g. Zwanzig and Liebermann^26^ documented a Silurian *Bohemoharpes* (Harpetidae, Trilobite) that utilised a nautiloid conch as refuge for moulting twice, as based on the obviously different dimensions between the cephalon and lower lamella, as well as the similar pattern of injuries at the brim of two sclerites. However, small protaspis or meraspis of *Omegops* has not been encountered in our collections. The almost uniform size of the cephala and thoraces supports that at least 7 *Omegops* moulted in the nautiloid conch. There is no obvious time gap between the moulting of different trilobites indicated by the superimposition and crowded manner of the exuviae. Possibly latter *Omegops* moulted in the conch shortly after the former completed ecdysis and left (for example after a few minutes, hours or days).

In summary, we propose the formation of this unusual specimen as follows: the first *Omegops* completed ecdysis and left, discarding the exuviae in the nautiloid living chamber simultaneously, then other *Omegops* sequentially or intermittently entered the living chamber to repeat this procedure, and the previous exuviae will then be buried by sea-floor sediments during this process. With time, more and more exuviae accumulated in the living chamber due to repeated ecdysis. However, the living chamber was a restricted communal space, and when there is not enough room for moulting, the latter *Omegops* may have cleared the former exuviae to the periphery to ensure they have sufficient space for moulting, thus leading to more sclerites preserved near the septum and conch-wall than those in the central part, as well as the scattered distribution of exuviae (i.e. disarticulated cephala, pygidia and thoracic segments) and some inversed sclerites (Fig. 3).

### Survival strategy and individual and/or social behaviours of trilobites in the middle Palaeozoic

In the middle Palaeozoic, marine prey suffered substantial predation pressure from hunting carnivores, such as fish, large arthropods and cephalopods^27^. The trilobites in this period could not escape such fate either. Fish^28^ and ammonoids^29^ were main predators of *Omegops* in the Late Devonian sea in the northwestern margin of the Junggar Basin. Moreover the study area is located in the oceanic island arc setting^30^, with violent volcanic activities during the Late Devonian to Early Carboniferous^31^. *Omegops* in our study lived in an unstable environment of rising sea level from the Frasnian to middle Famennian in the northwestern margin of the Junggar Basin^32^, and calcareous tempestites are widespread; these environmental factors may also have influenced trilobites in seeking shelter, and forced them to employ the hiding-for-moulting strategy.

The diversity and abundance of organisms were greatly reduced after the F-F mass extinction event in most areas of the world^33^. By contrast, as a refuge for avoiding the adverse effects of the F-F event, the study area is typified by abundant and diversified faunas instead of recession^34^. Various groups of organisms flourished vigorously in the same ecological niche with *Omegops*, which were bound to cause a decline in the living space of *Omegops* and increasing competition pressure. Therefore, in the context of the powerful predation pressure, unstable marine environments and competition pressure of organisms occupying the same ecological niche, *Omegops* may have developed a survival strategy of moulting in the nautiloid conch, an adaptive evolution caused by above adverse factors. After moulting the new individuals are usually soft-shelled for most arthropods, including trilobites^13^. As an example, the extant aquatic arthropods (Crustacea: *Eriocheir sinensis*) will crouch down next to the exuviae waiting for hardening for some time^35^. Probably this phacopid trilobite may have adopted similar behaviours; under the powerful predation and competition pressures, soft-shelled trilobites would be easier to be hurt due to the interferences of other organisms, whereas hiding in the nautiloid conch can avoid such interferences. In this way, the nautiloid conch can act as a temporary habitat for trilobites (Fig. 3).

As a result of ecdysis, at least 7 individual trilobites were preserved in the same conch, which implies a likely social behaviour of trilobites. As previously mentioned, it is unlikely that 7 *Omegops* moulted simultaneously, namely, there is a few minutes, hours even days after the former has completed ecdysis and left, before the latter trilobite enters the conch to repeat this process. The discarded exuviae in the conch may give a cue of “safety for moulting” for the latecomers, thus attracting trilobites in the same area to choose the same location for moulting. In conclusion, the hiding and herding moulting strategy suggests that phacopid trilobites had their own survival skills and behavioural habits in the middle Palaeozoic.

## Conclusions

Autochthonously preserved and undamaged sclerites of *Omegops* emplaced in a nautiloid living chamber from the Upper Devonian of the northwestern margin of the Junggar Basin are interpreted as exuviae, rather than corpses of *Omegops*, food residue of nautiloids or mechanical aggregation by post-mortem transportation. There are at least 7 *Omegops* individuals that moulted in the nautiloid living chamber and left disarticulated exoskeletions there. The remarkable accumulation of trilobite exuviae rules out the likelihood that the fossil aggregation was resulted from sedimentary events. The exuviation of phacopid trilobites within the nautiloid conch is the embodiment of the cryptic behaviour, which results from adaptive behavioural evolution caused by the complex and unstable marine ecological environments during the middle Palaeozoic. A number of trilobites moulting in the same ‘haven’, may imply a social behaviour, shedding a new light on the collective survival strategies of the middle Palaeozoic trilobites.

### Geological setting and method

The Upper Devonian is composed of two parts in the northwestern margin of the Junggar Basin: the lower part is continental strata of the Frasnian Zhulumute Formation, and the upper part represented by the marine strata of the Hongguleleng Formation. The Hongguleleng Fm. being subdivided into three members^36^, is primarily Famennian in age, but the F-F (Frasnian-Famennian) boundary may exist at the bottom^32^. In contrast to the barren biota after F-F mass extinction event in the rest of the world, high abundance and diversity of body fossils are seen in the Famennian strata of the Hongguleleng Formation, even enriched layers of macrofossils. Therefore, this area is probably a refuge for escaping the influences of the F-F mass extinction event^34^.

Trilobites are principally concentrated in the upper part of the lower and middle members of the Hongguleleng Formation with a high abundance but lower diversity. There are only 3 species of Phacopidae, including *Omegops accipitrinus mobilis*, *O. cornelius* and *Phacops circumspectans tuberculosus*^37^,^38^. Apart from some enrolled and articulated exoskeletons which were corpses, most of these trilobites are scattered sclerites. Some thoraces articulated with pygidia, but with cephala nearby, are identified here as exuviae. In other cases, cephala, thoraces and pygidia are separated from each other, including some isolated thoracic segments. The preservation indicates that the corpses or exuviae of trilobites might have been carried by seawater before they were finally buried.

**Figure 1:**
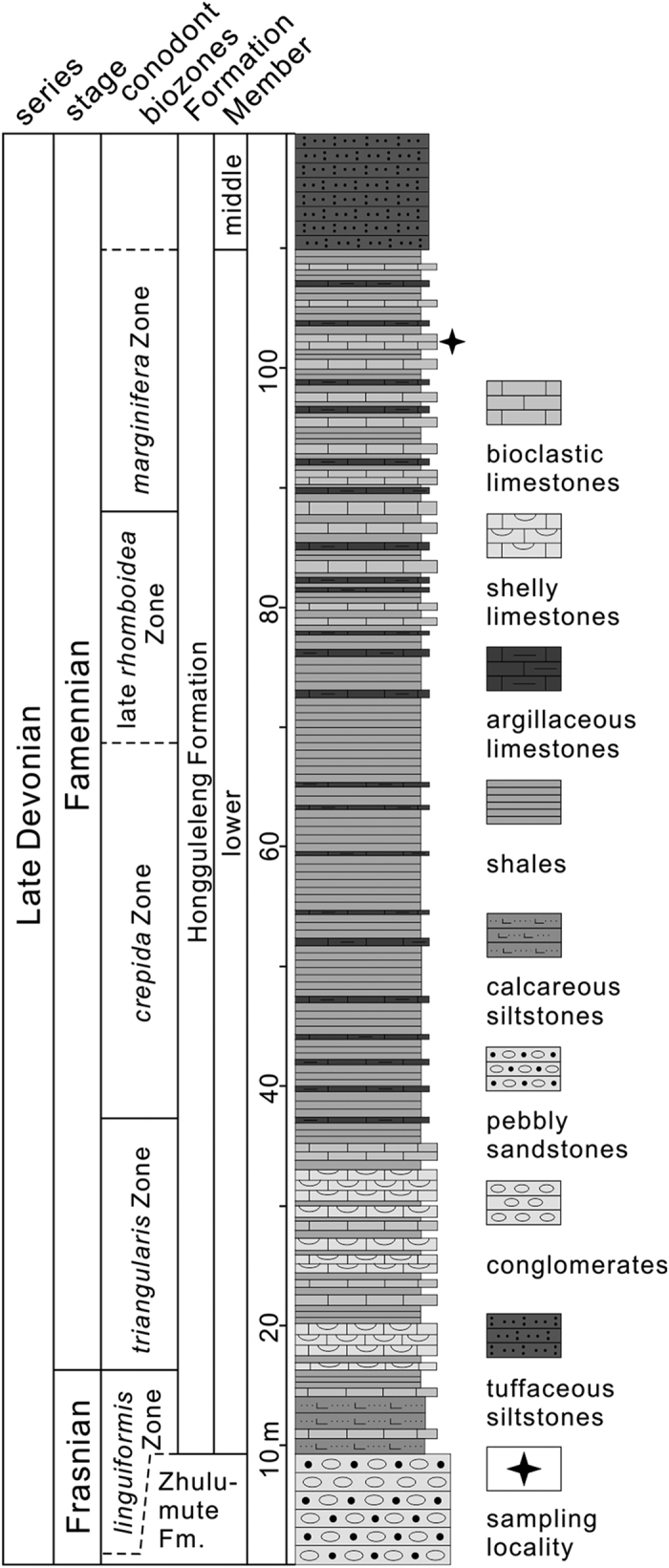
Sketch showing the sampling locality and the stratigraphic column of the lower Member of the Hongguleleng Formation. (conodont biozones after Suttner *et al*.^32^, drawn by R.-W. Z. using the CorelDRAW X5 software, Copyright (c) 2016 R.-W. Z. and its licensors. All rights reserved).

**Figure 2:**
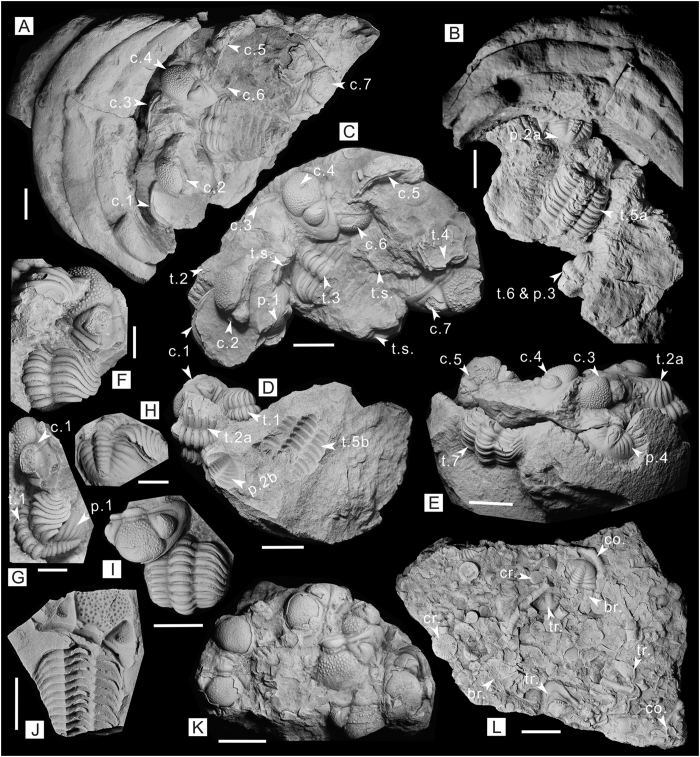
Nautiloid containing disarticulated exoskeletons of *Omegops cornelius*, as well as trilobites and bioclastic limestones outside the conch. **(A–E)** The distribution of the sclerites of *Omegops* in the nautiloid living chamber (HB2013-01), figure B presenting the status after the specimen in figure C was stripped from the previous specimen in figure A, figure D is the reverse side of the specimen in figure C, figure E showing the sclerites of *Omegops* near the septum after 4 septa were stripped from the previous specimen. **(F–H)** The exuviae of *Omegops* in the living chamber, showing the dorsal view, side view and back view, respectively. **(I,J)** The exuviae of *Omegops* outside the conch from the argillaceous limestones of the Hongguleleng Formation, figure I (HB-04) showing the enrolled thorax and pygidium, but with cephalon nearby, figure J (HB2013-05) showing the cephalon situated in the right-anterior of thorax with distinct rotation. **(K)** The cluster of cephala of *Omegops* outside the conch from the bioclastic limestones of the Hongguleleng Formation, and all cephala are preserved dorsal side upward (HB213-04). **(L)** Bioclastic limestones of the storm bed, showing the sclerites of *Omegops* buried together with crinoids, bryozoans, corals and other fossils (HB2013-02). c. = cephalon, t. = thorax, t.s. = thoracic segment, p. = pygidium, tr. = trilobite, co. = coral, cr. = crinoids, br. = bryozoans. The scales of figures (**F–H)** are 5 mm, other scales are 10 mm. (Photographs taken by R.-W. Z.).

**Figure 3:**
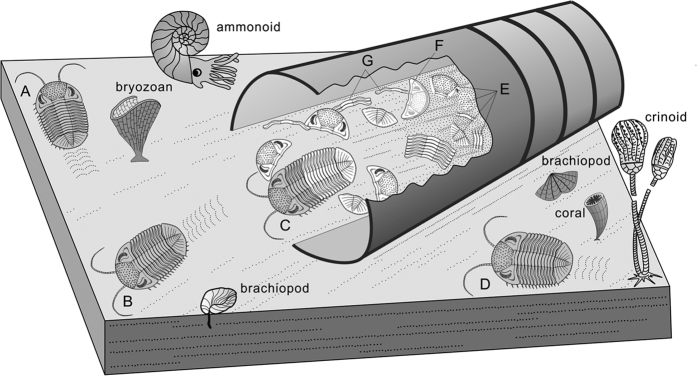
Reconstruction of *Omegops* moulting within the nautiloid living chamber. **(A**) *Omegops* has completed moulting and moved away from the nautiloid. **(B)** *Omegops* leaving the nautiloid after moulting. **(C)** *Omegops* moulting within the living chamber. **(D)** *Omegops* moving outside the living chamber normally. **(E)** Previous exuviae are buried gradually by sediments. **(F,G)** Previous exuviae are moved to the edge of the nautiloid by *Omegops* preparing for moulting, inducing overturned cephalon (**F**) and scattered thoracic segments (**G**). (drawn by R.-W. Z. using the CorelDRAW X5 software, Copyright (**c**) 2015–2016 R.-W. Z. and its licensors. All rights reserved).

The trilobites described in this paper were collected from the upper part of the lower Member of the Hongguleleng Formation in the Boulongour section, 30 km north of Hoxtolgay Town, Hoboksar, NW Xinjiang. The upper part of the lower Member is composed of argillaceous limestones, bioclastic limestones and shales (Fig. 1), which were deposited in an environment with the distal influence of storms. This unusual specimen was collected from the bioclastic limestones (storm bed) yielding abundant brachiopods, crinoids, corals, bryozoans, gastropods, cephalopods and other macrofossils, which is equivalent to the conodont *P. marginifera* Zone of the Late Devonian Famennian^32^. All specimens are housed in the State Key Laboratory of Biogeology and Environmental Geology, China University of Geosciences (Wuhan). The trilobites were prepared using mechanical techniques, including pneumatic air scribe and needles under a microscope, and all photographs in Fig. 2 were taken using a Nikon D5100 camera with a Micro-Nikkor 55 mm f2.8 lens after the fossils whitened with magnesium powder.

## Additional Information

**How to cite this article**: Zong, R.-W. *et al.* Seven 365-Million-Year-Old Trilobites Moulting within a Nautiloid Conch. *Sci. Rep.* **6**, 34914; doi: 10.1038/srep34914 (2016).
